# Metabolic Crossroad Between Macrophages and Cancer Cells: Overview of Hepatocellular Carcinoma

**DOI:** 10.3390/biomedicines12122684

**Published:** 2024-11-25

**Authors:** Anna Santarsiero, Paolo Convertini, Dominga Iacobazzi, Vittoria Infantino, Simona Todisco

**Affiliations:** 1 Department of Health Sciences, University of Basilicata, 85100 Potenza, Italy; anna.santarsiero@unibas.it (A.S.); vittoria.infantino@unibas.it (V.I.); 2 Department of Basic and Applied Science, University of Basilicata, 85100 Potenza, Italy; paolo.convertini@gmail.com; 3 Bristol Medical School, Translational Health Sciences, University of Bristol, Bristol BS2 8HW, UK; domingaiacobazzi@live.it

**Keywords:** macrophages, hepatocellular carcinoma, cancer cells, cellular signals, mitochondria, metabolic reprogramming

## Abstract

The metabolic interplay between macrophages and cancer cells mirrors the plasticity of both kinds of cells, which adapt to the microenvironment by sustaining cell growth and proliferation. In this way, cancer cells induce macrophage polarization, and, on the other hand, tumor-associated macrophages (TAMs) contribute to the survival of cancer cells. In a simplified manner, macrophages can assume two opposite subtypes: M1, pro-inflammatory and anti-tumor phenotype, and M2, anti-inflammatory and protumor phenotype. How do cancer cells induce macrophage polarization? Any actor involved in tumor growth, including the mitochondria, releases molecules into the tumor microenvironment (TME) that trigger a subtype transition. These metabolic changes are the primary cause of this polarization. Hepatocellular carcinoma (HCC), the prevalent type of liver primary tumor, is characterized by cells with extensive metabolic adaptions due to high flexibility in different environmental conditions. This review focuses on the main metabolic features of M1 and M2 macrophages and HCC cells underlying their metabolic behavior in response to TME.

## 1. Introduction

Hepatocellular carcinoma (HCC) is the most prevalent type of primary tumor of the liver. Liver cancer ranks as the sixth most frequently diagnosed cancer and the third leading cause of cancer-related deaths worldwide [1]. In 2022, nearly 865,000 new cases were diagnosed, and the disease was responsible for 760,000 deaths [2]. The high mortality rate of liver cancer is largely due to its aggressive nature, late-stage diagnosis, and association with chronic liver conditions, such as cirrhosis and hepatitis B (HBV) or hepatitis C (HCV) infections, which are responsible for the highest percentage of HCC cases [1]. It is worth noting that HCC develops in 80% to 90% of individuals with cirrhosis [3]. Additional risk factors include aflatoxin exposure, heavy alcohol consumption, excess body weight, smoking, and many diseases [4]. It has become increasingly established that metabolic dysfunctions play a fundamental role in the initiation and progression of HCC [5]. As a matter of fact, despite the declining rates of chronic hepatitis infections, the incidence of new HCC cases is on the rise and is projected to increase by 55% between 2020 and 2040 [1,6]. This trend can be attributed to the rising prevalence of metabolic disorders, such as metabolic-associated fatty liver disease (MAFLD), metabolic syndrome, type 2 diabetes, and obesity.

Tumors develop a distinctive metabolic profile influenced by numerous factors, such as poor tissue perfusion, nutrient competition, and the accumulation of metabolic byproducts. Neoplastic cells are especially known for preferring glycolysis over oxidative phosphorylation, even in the presence of adequate oxygen—an adaptation known as the Warburg effect [7,8]. HCC cells are characterized by extensive metabolic reprogramming to sustain their growth and survival with high flexibility in responding to environmental conditions [9]. Transformed hepatocytes display increased glycolysis and lactic acid production, together with mitochondrial metabolism and tricarboxylic acid (TCA) cycle rewiring, which plays a main role in ensuring the crosstalk between mitochondria and cytosol as well as participating in the trafficking of signal molecules between cells and the microenvironment [4,10].

The tumor microenvironment (TME) is a dynamic and intricate ecosystem in which cancer cells coexist with different non-malignant cells, all embedded within a vascularized extracellular matrix. The TME encompasses a wide range of immune cells, each with specific phenotypes and lineages, along with diverse stromal cells, including cancer-associated fibroblasts, mesenchymal stem cells, tumor-associated adipocytes, tumor endothelial cells, and pericytes [11]. In addition, the TME comprises an extracellular component made up of soluble proteins, the extracellular matrix, and essential nutrients (i.e., glucose and amino acids) [12]. Interestingly, the crossroad between cancer cells and TME cells leads to the remodeling of the metabolic answers of all cells involved in a new co-life to promote the survival of cancer cells [13]. In this context, TME functions to store nutrients, metabolites, such as lactic acid, and signal molecules released from cancer cells. At the same time, cancer cells need metabolites and signal molecules to induce TME cells to cooperate with them.

Tumor-associated macrophages (TAMs) represent a heterogeneous and plastic fraction of immune cells in TME whose metabolism is significantly reprogrammed to participate in building a “tumor” equilibrium with a metabolic adaptation. Indeed, in response to inflammatory stimuli, macrophages adopt an M1-like phenotype that produces inflammatory cytokines, while anti-inflammatory signals lead to an M2-like phenotype that is immunosuppressive. Tumorigenesis is characterized by pro-inflammatory M1-like macrophages that release inflammatory mediators; however, when the tumor is established, TAMs shift toward an M2-like phenotype by promoting chronic inflammation, which favors tumor development. Once established, the M2-like macrophages are predominantly present in malignant tumors, contributing to tumor growth, angiogenesis, and metastasis [14]. Moreover, M2-like TAMs may play a role in drug resistance and enhance cancer stemness in HCC, leading to a worse prognosis for patients [15]. Lactate secreted by HCC cells facilitates interactions between TAMs and cancer cells, further promoting the M2-like phenotype [16]. Immunometabolic plasticity is a main feature of macrophages exploited by tumor cells for their growth and development in order to create a self-sufficient cell community. This review will focus on the crosstalk between macrophages and HCC cells for metabolic reprogramming and functional adaption.

## 2. Macrophages—Origin

In vertebrates, the immune system is responsible for the organism’s defense and is organized in the innate immune system, present at birth, and the adaptive immune system, developed when the body is exposed to microorganisms or chemicals.

The innate immune theory was formulated around 1892 from the observations of Metchnikoff regarding the larvae’s ability to eliminate foreign particles from their bodies by using “non-digestive” cells defined as phagocytes (from Greek phagein, “eat”, and cytos, “cells”) in a process called phagocytosis [17]. The theory of immunity of Metchnikoff was opposed to the humoral theory of immunity based on antibody ability to destroy invading microorganisms, which prevailed until 1940 when both theories were considered to define two important aspects of immunity: innate immunity and adaptive immunity response [18]. When pathogens or foreign substances are introduced into the organism, the first response is the quick activation of the immune system cells that try to eliminate them. This is a nonspecific physical, chemical, and cellular response characterized by the production of different “exogenic” or “endogenic” molecules. The innate response in vertebrates, including humans, is the first line of defense against the entry of external microorganisms into the body through the production of various molecules, including mucous or hydrochloric acid. The second line of defense is the production of endogenous molecules, as well as cytokines or interferons, as a strategy to confine the external insult and resolve the invasion [19].

Among the different cell types involved in immune response, macrophages (from Greek makròs, i.e., “big”, and phagein, i.e., “eat”) help initiate specific defense mechanisms by recruiting other immune cells such as lymphocytes. Macrophages are classified into circulating macrophages and resident-tissue macrophages. Initially, it was believed that all macrophages originated from circulating blood monocytes, which were derived from progenitors in the bone marrow, and that they were rapidly dividing cells able to quickly migrate to the injury site [20,21]. These observations led to the formulation of the mononuclear phagocyte system (MPS) hypothesis in which promonocytes, derived from bone marrow, differentiate in monocytes in the peripheral blood to generate macrophages in tissues [22]. Subsequent evidence underlined that resident-tissue macrophages can proliferate within the tissue. It was discovered that a macrophage population can originate from the yolk sac but can also derive from both embryonic and adult stem cells [23]. Finally, the function of resident-tissue macrophages is closely related to the tissue in which they are found [24].

Currently, resident tissue macrophages are considered a kaleidoscope of different progenitors that adopt various phenotypes and functions, thereby modulating a dynamic tissue response [25] and contributing to tissue homeostasis [26]. It is unknown, at this time, whether resident tissue-derived macrophages and circulating blood monocyte-derived macrophages belong to distinct subpopulations or if their function varies depending on the cellular microenvironment.

## 3. Macrophage Classification and Function

Macrophages are localized in all tissues, including the lung, spleen, liver, brain, kidney, skin, heart, and intestinal tract [27]. They have different shapes depending on the tissue: for example, Kupffer cells, which are localized in liver sinusoids, have a star-like shape, while microglia, which are resident immune cells of the central nervous system (CNS), exhibit morphological changes from ramified cells to amoeboid cells during activation [28,29].

Under normal conditions, macrophages display a “resting state”, whereby their metabolism ensures the “normal” cellular response; this condition is often referred to as unpolarized macrophages or M0. However, they can also rapidly undergo reprogramming in response to microenvironmental stimuli, switching into an “activated state”. This macrophagic rearrangement, defined as polarization, results in the formation of “classically activated M1” or “alternatively activated M2” macrophages [26].

The M1 polarization is driven by external factors, such as pathogen-associated molecular patterns (PAMPs) like bacterial lipopolysaccharide (LPS) [30], as well as internal substances like the cytokine interferon-ɣ (IFN-γ) [31].

Other molecules or factors that induce M1 polarization are the interferon regulatory factor (IRF) and suppressor of cytokine signaling (SOCS) [32]. The IRF is a family of nine intracellular proteins (IRF1-9), and IRF1, IRF5, and IRF8 regulate macrophage maturation and M1 polarization [33]. SOCS is a family of eight cytokine-inducible proteins (CIS and SOCS1–SOCS7). In particular, SOCS3 is the isoform involved in M1 polarization that shows a low expression in resting-state macrophages but an increased expression in classical activated macrophages [34]. SOCS3 inhibits the activity of Janus kinase (JAK), a tyrosine kinase protein that controls immune response and hematopoiesis. SOCS3 also promotes nitric oxide (NO) production via inducible nitric oxide synthase (iNOS) [34] and controls nuclear factor-κB (NF-κB) involved in the M1 activation [35].

Overall, M1 macrophage activation can be described as a “signal loop” response, where M1 macrophages produce pro-inflammatory cytokines like TNF-α that sustain their own activation.

The “alternatively activated M2” macrophages are categorized into subtypes: M2a, M2b, M2c, and M2d, based on the different stimuli that induce their polarization [36]. Recently, other macrophage subtypes have been identified, such as Mox macrophages, which are activated by oxidized phospholipids (OxPL) [37], M4 macrophages, activated by chemokine ligand 4 (CLCX4) [38], and Mhem macrophages, activated by hemoglobin (Hb) and present in atherosclerotic regions [39]. In Table 1, macrophage types and subtypes and their stimuli are indicated.

## 4. Link Between the Metabolism of Macrophages and HCC Cells

The metabolism of M1 and M2 macrophages reflects their distinct functions. M1 macrophages primarily rely on aerobic glycolysis, even in the presence of oxygen. Although this metabolic pathway is less efficient in terms of ATP production, it provides rapid energy and the intermediates necessary for biosynthetic processes, such as the synthesis of nucleotides, amino acids, and lipids. M1 macrophages also exhibit increased activity in the pentose phosphate pathway (PPP), which contributes to NADPH production and reactive oxygen species (ROS) generation. In contrast, M2 macrophages rely predominantly on oxidative phosphorylation (OXPHOS) and fatty acid oxidation (FAO). This metabolism guarantees more efficient ATP production and supports long-term functions necessary for tissue repair and anti-inflammatory responses [40].

Macrophages can rapidly switch between the M1 and M2 phenotypes through significant metabolic shifts. This plasticity enables macrophages to adapt quickly to changing microenvironmental cues, ensuring that appropriate immune responses are mounted according to the physiological needs.

Interestingly, many cancer cells, including HCC cells, exhibit metabolic behavior similar to M1 macrophages, particularly the reliance on aerobic glycolysis. This metabolic imprinting of macrophages is hypothesized to support the rapid proliferation of cancer cells by providing the necessary biosynthetic precursors for cell growth and division.

### 4.1. Glucose Metabolism

Glucose is the main energy source for many cells, which depend on its availability to sustain their health.

LPS-stimulated macrophages and many kinds of cancer cells, including HCC cells, exhibit elevated glycolysis. This increase is associated with the enhanced expression of glycolytic enzymes, regulated by hypoxia-inducible factor 1 α (HIF-1α) [41,42]. In particular, the isoform 1 of glucose transporter, GLUT1, hexokinase (HK), phosphofructokinase 2 (PFK2), and pyruvate kinase (PK) were found to be overexpressed in cancer cells [41] and in macrophages [40].

**Figure 1:**
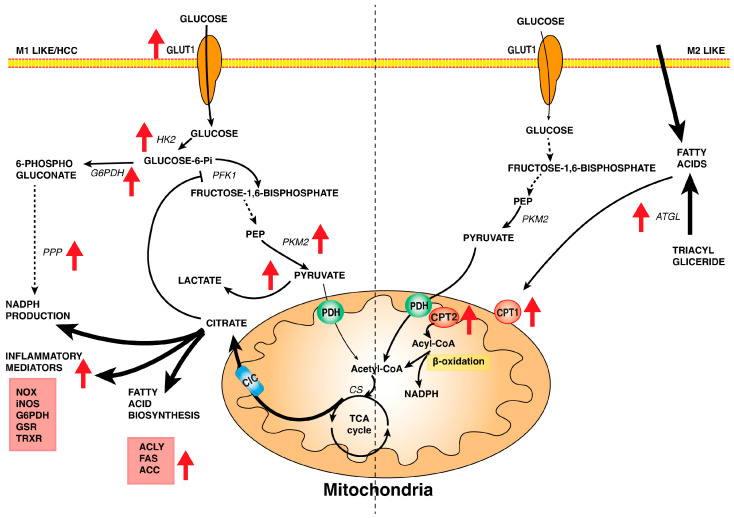
The fate of glucose and fatty acids in macrophages and HCC cells. By starting from the transport of glucose and fatty acids, metabolic differences in M1 macrophages/HCC cells vs. M2 macrophages are shown, underlining the role of mitochondria. In M1 macrophage/HCC cells, the glucose uptake increases for the overexpression of GLUT1. Glucose is converted by overexpressed HK2 into Glucose 6–phosphate, which is addressed toward glycolysis, supplying energy to cells, and the pentose phosphate pathway to provide NADPH for the biosynthesis of the fatty acids and inflammatory mediators. Indeed, many enzymes of both pathways are upregulated as PKM2 for glycolysis and G6PDH for PPP. The fate of pyruvate is to produce lactate by the upregulated LDHA or acetyl-CoA through pyruvate dehydrogenase (PDH). Acetyl-CoA takes part in the interrupted TCA cycle, leading to an increase in mitochondrial citrate. The citrate is transported by CIC into the cytosol, where it participates in the biosynthetic pathways. In M2 macrophages, glucose transport and glycolysis are not the primary pathways, whereas the uptake of fatty acids and the triacylglyceride mobilization increased. The β-oxidation of fatty acids is the predominant metabolic pathway; indeed, there is an upregulation of CPT1 and CPT2. Abbreviations: GLUT1—glucose transporter isoform 1; HK2—hexokinase 2; PFK1—phosphofructo kinase 1; PEP—phosphoenolpyruvate; PKM2—pyruvate kinase M2; LDHA—lactate dehydrogenase A; PPP—pentose phosphate pathway; NOX—NADPH oxidase; iNOS—inducible nitric oxide synthase; G6PDH—glucose-6-phosphate dehydrogenase; GSR—glutathione-disulfide reductase; TRXR—thioredoxin reductase; ACLY—ATP citrate lyase; FAS—fatty acid synthase; ACC—acetyl-CoA carboxylase; ATGL—adipose triglyceride lipase; PDH—pyruvate dehydrogenase; CS—citrate synthase; CIC—citrate/isocitrate carrier; CPT1 and CPT2—carnitine palmitoyltransferase isoform 1 and 2; TCA cycle—tricarboxylic acid cycle. Red arrows indicate enzymes or pathways that are upregulated.

GLUT1 shows a high affinity for glucose (Km about 1 mM) and ensures that it is transported effectively. Glucose triggers the macrophage metabolic response since it is “trapped” as glucose-6-phosphate by HK in the cells. HK2, the higher affinity isoenzyme, is the isoform upregulated in M1 macrophages [40] and replaces glucokinase (GCK) in HCC cells [43,44]. Furthermore, M1 macrophages express uPFK2, the most active isoform of phosphofructokinase 2 (PFK2), encoded by the PFKFB3 gene, which has a higher kinase activity to synthesize fructose 2,6-bisphosphate, the main activator of phosphofructokinase 1 (PFK1), resulting in the glycolysis activation and an enhanced glycolytic flux [40]. Also, in cancer cells and HCC cells, the PFKFB3 gene was found to play a significant role in the activation of glycolysis (Figure 1) [45].

PK, involved in the final step of glycolysis, has an interesting role in M1 macrophages, as well as in cancer cells [46]. PKM2 is the upregulated isoform in LPS-triggered macrophages and is responsible for the switching from oxidative phosphorylation to aerobic glycolysis. Likewise, in cancer cells, as well as HCC cells, PKM2 is upregulated, and its expression is related to poor prognosis [47]. More in detail, a total of 16 studies with 18 cohorts used for meta-analysis reveal a correlation between PKM2 expression and clinicopathological features in solid tumors of the digestive system and indicate that high PKM2 levels are associated with poor overall survival [48]. Furthermore, a study performed with HCC tissues and adjacent non-tumor liver tissues from 688 HCC patients subjected to curative resection demonstrates a PKM2 overexpression in the presence of high tumor node metastases, vascular invasion, and shorter overall survival [49]. Interestingly, in this condition, PKM2 is not only a metabolic enzyme, but it also acts as a regulatory enzyme that translocates in the nucleus and interacts with HIF-1α by inducing the overexpression of GLUT1, lactate dehydrogenase (LDHA), and pyruvate dehydrogenase kinase 1 (PDK1), the regulatory enzyme of pyruvate dehydrogenase (PDH), as well as the expression of pro-inflammatory cytokines, including IL-1β [50] or VEGF-A, promoting growth and angiogenesis [43].

Tumor cells are characterized by aerobic glycolysis, known as the “Warburg effect”, in which a large amount of glucose, converted into pyruvate, is addressed to lactate accumulation. This metabolic reprogramming of tumor cells has been found to increase in HCC [51], and it was demonstrated, by NMR-based metabolomic analysis, that the serum of HCC patients showed a higher lactate concentration compared to normal subjects [52].

Lactate is transported out of the cells by the monocarboxylate transporters (MCTs) [53], particularly by MCT1, MCT2, and MCT4 isoforms whose expression was found altered in cancer cells from patients affected by different cancer types [54]. MCT4 was found overexpressed and was related to cell proliferation, tumor cell invasion, and poor prognosis in HCC patients [55].

Extracellular lactic acid affects TME. In fact, TAMs undergo a shift toward the M2 phenotype dependent on the stabilization of HIF-1α that activates NF-κB signaling and increases transcription of genes encoding VEGF, IL-8, and Arg1 by promoting angiogenesis and M2 polarization [16].

This underlines that the metabolism can drive the gene expression to sustain metabolic changes and address the macrophagic response and cancer proliferation.

In M2 macrophages, glycolysis is not the hub of macrophage activation; in fact, the M2 differentiation is not affected by glucose depletion [56]. Furthermore, after macrophage stimulation by IL-4, the expression of GLUT1 was found unchanged, while L-PFK2 isoform was found to be overexpressed. L-PFK2 has an increased bisphosphatase activity that leads to a decrease of fructose 2,6-bisphosphate, followed by a reduction of glycolytic flux [57] (Figure 1).

In conclusion, although glycolysis is required to sustain the macrophage M2 activation, it is not the main metabolic pathway that drives the polarization shift [58]. In LPS-activated macrophages and different cancer cells, the PPP plays a relevant role in ensuring the production of NADPH and the synthesis of nucleic acids, and some enzymes of oxidative and non-oxidative phases of the PPP were found to be activated. Particularly glucose 6-phosphate dehydrogenase (G6PDH), the first and rate-limiting enzyme of the oxidative phase of PPP, was overexpressed [59,60] (Figure 1). G6PDH synthesizes NADPH, which is used for the biosynthesis of fatty acids, PUFA, and prostaglandins. NADPH is the cofactor of NADPH-oxidase (NOX), which is involved in ROS synthesis in macrophages, but also the cofactor of detoxification systems, such as glutathione reductase (GSR) or thioredoxin reductase (TRXR), critical in HCC cells. Therefore, it could be interesting to investigate the different fate of the PPP NADPH pool, which could be directed to control the redox balance or to synthesize lipids, prostaglandins, and signal molecules in activated macrophages and cancer cells.

On the other end, M2 macrophages have a limited flux through the PPP [61]. IL-4 activation leads to the expression of the carbohydrate kinase-like protein CARKL, which catalyzes the synthesis of sedoheptulose-7-phosphate related to low glycolytic rates.

### 4.2. Lipid Metabolism

Lipid metabolism is a hallmark of macrophage function, and the shift between lipid biosynthesis and β-oxidation of lipids is related to M2 macrophage polarization. Lipid metabolism is at the crucial crossroad between the need for cells to produce a large amount of ATP through fatty acid β-oxidation and the TCA cycle in the mitochondria and the accumulation of lipids for proliferation and cell growth via lipid biosynthesis.

The accumulation of lipids is a hallmark of M1 macrophages and HCC cells, whereby lipid biosynthesis is relevant to address the immunometabolism response in M1 macrophages and cancer cells [41].

Conversely, the fatty acid β-oxidation is the predominant metabolic pathway in M2 macrophages.

In particular, M1 macrophages and HCC cells are characterized by the overexpression or activation of many enzymes involved in lipid biosynthesis and inflammatory response. Notably, enzymes such as ATP citrate lyase (ACLY) citrate carrier (CIC), NADPH oxidase (NOX) [62], acetyl CoA carboxylase (ACC) [63], and fatty acid synthase (FAS) [64] play crucial roles in driving pro-inflammatory response and were found to be overexpressed in HCC cells [4,65,66] (Figure 1). In contrast, M2 macrophages activate the carnitine shuttle via carnitine palmitoyl transferase 1 (CPT1), which, in the murine macrophage cell line, contributes to the regulation of the inflammatory response [67]. Adipose triglyceride lipase (ATGL), an enzyme involved in triglyceride metabolism, is related to the M2/M1 macrophage switch. Thus, LPL-induced ATGL inhibition results in decreased lipolysis of triglycerides and increased accumulation of fatty acids [68], as well as the generation of various bioactive molecules that contribute to the regulation of biological processes (Figure 1).

One of these bioactive molecules, prostaglandin E2 (PGE2), is produced from arachidonic acid via cyclooxygenase (COX) and prostaglandin E synthase (PGES). Although PGE2 can be secreted by almost all cell and tissue types, it is related to the function of M1-like macrophages, and it is the main prostaglandin produced in HCC as a key factor of HCC tumorigenesis [69].

Interestingly, PGE2 induction was related to the macrophage reprogramming with polarization from M1-like to M2-like macrophages via EP4 receptors and subsequent activation of cAMP response element-binding protein (CREB) signaling, the main transcriptional factor associated with M2 macrophages. On the contrary, the inhibition of PGE2 synthesis results in an anti-tumor effect and changes in macrophage functions [70].

Many transcription factors are involved in addressing the lipid metabolism in activated macrophages. In M1 macrophages, fatty acid biosynthesis is under the control of sterol regulatory element-binding proteins (SREBPs). Particularly, the isoform SREBP-1a regulates the expression of two enzymes of de novo lipogenesis, ACC and FAS [71]. Interestingly, the expression of SREBP-1 is increased in HCC tissues, and SREBP-1 knockdown suppresses the proliferation and migration of HCC cells [72].

NF-κB is a pivotal M1 macrophage transcription factor whose full activation is also under the control of ACLY. Once activated, NF-κB fosters the transcription not only of classical pro-inflammatory genes but also of genes encoding lipid metabolism proteins such as CIC and ACLY [73]. Interestingly, SREBP1 is induced by NF-kB in activated macrophages [74]. Additionally, in cancer cells, NF-kB signaling is an important regulator of cell growth and proliferation and its inhibition was found to suppress HCC growth [75]. Among the peroxisome proliferator-activated receptors (PPARs), peroxisome proliferator-activated receptor-γ (PPARγ) drives the activation of fatty acid oxidation in M2 macrophages [76]. Mechanistically, lipid metabolism is finely tuned through different posttranscriptional factors, including microRNAs (miRNAs) in macrophages. In this context, miR-33 has been implicated in regulating SREBPs, and inhibitors of miR-33 suggest an anti-atherosclerotic effect [77].

### 4.3. Amino Acid Metabolism

#### 4.3.1. L-Arginine Metabolism

L-arginine levels affect the metabolism of cancer and immune cells. L-arginine is involved in macrophage function, and its fate seems to be different for M1 and M2 macrophages. Furthermore, high levels of L-arginine promote tumor growth via metabolic rewiring and transcriptional regulation. The increased levels of HCC are due to enhanced absorption and decreased conversion to polyamines [78].

L-Arginine is the substrate of iNOS to synthesize NO, an important inflammatory mediator in M1 macrophages, while in M2 macrophages, L-Arginine is utilized by arginase enzymes (ARG1 and ARG2) for the biosynthesis of proline and polyamines. iNOS presents a Km value for L-arginine of about 5 µM, whereas arginase Km is about 10 mM. However, the Vmax of arginase is about 1000-fold higher than that of iNOS [79]. Considering these kinetic characteristics, it can be speculated that the L-arginine pool homeostasis could be essential to address the L-arginine metabolism.

L-arginine can be derived either from the external uptake by different members of the solute carrier family 7 or via internal de novo biosynthesis from ornithine. The identified isoforms involved in L-arginine uptake are SLC7A1, the constitutive isoform, SLC7A2, the inducible isoform, and SLC7A3, widely expressed during embryonic development [80].

**Figure 2:**
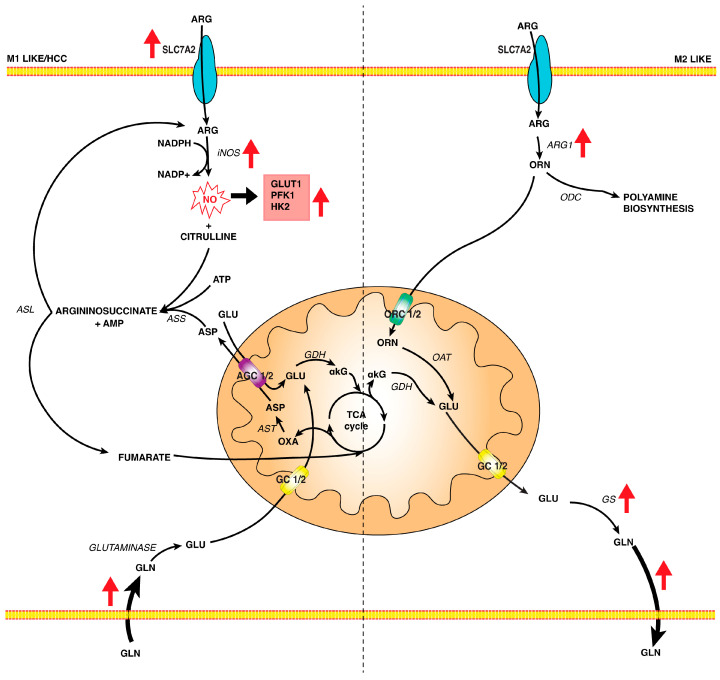
Crossroad in amino acid metabolism in macrophages and HCC cells. Metabolism of L-arginine and L-glutamine in M1 macrophages/HCC cells and M2 macrophages and mitochondrial function. In M1 macrophages and HCC cells, both L-arginine transporter and iNOS are overexpressed. The increased level of NO contributes to the activation of glycolytic enzymes, whereas citrulline produces fumarate, which supplies the interrupted TCA cycle. Furthermore, the increased L-glutamine uptake leads, via glutaminase, to L-glutamate, which takes part in the TCA cycle, supplying the energy needs of the cell and sustaining arginine metabolism through aspartate recycling. In M2 macrophages, L-arginine is converted into L-ornithine by overexpressed ARG1. L-ornithine is the substrate for the polyamine biosynthesis into the cytosol and produces L-glutamate in mitochondria through OAT. Upregulated GS transforms L-glutamate into L-glutamine, which HCC cells use as a carbon source. Abbreviation: ARG—L-arginine; SLC7A2—arginine transporter; iNOS—inducible nitric oxide synthase; NO—nitric oxide; GLUT1—glucose transporter isoform 1; PFK1—phosphofructo kinase 1; HK2—hexokinase 2; ASS—argininosuccinate syntase; ASL—argininosuccinate lyase; ASP—L-aspartate; GLU—L-glutamate; GLN—L-glutamine; ARG1—arginase; ODC—ornithine decarboxylase; GS—glutamine synthetase; AGC1/2—aspartate/glutamate carrier isoform 1 and 2; GC 1/2—glutamate carrier isoform 1 and 2; OXA—oxalacetate; AST—aspartate aminotransferase; GDH—glutamate dehydrogenase; αKG—α-ketoglutarate; TCA cycle—tricarboxylic acid cycle; ORN—L-ornithine; ORC 1/2—ornithine carrier isoform 1 and 2; OAT—ornithine aminotransferase. Red arrows indicate enzymes or pathways that are upregulated.

The activation of macrophages by LPS leads to an increase in the uptake of L-arginine, with an overexpression of the SLC7A2 [81] and iNOS, which catalyzes the reaction of L-arginine with NADPH to produce NO and L-citrulline. NO acts as a signaling molecule in the inflammatory response and is also involved in the metabolic reprogramming of tumor cells. In fact, NO stimulates glycolysis via the upregulation of mRNA levels of GLUT1 and PFK1 [82] or via overexpression of HK2 [83] (Figure 2).

Many cancers, such as liver, breast, and colon, express NOS, but it is not clear if its effect can be classified as “pro-cancer” or “anti-cancer” [84]. Recently, it was demonstrated that the overexpression of iNOS in CD24 + CD133+ liver cancer stem cells of HCC patients is associated with worse outcomes [85]. Moreover, iNOS expression evaluated in slides from a stage III melanoma TMA is associated with poor survival [86]. Among the potential molecular mechanisms responsible for this effect, high levels of NO were found to foster cell migration and proliferation of primary human epidermal stem cells in a study performed by Zhang et al. [87].

According to a number of studies, persistent exposure to NO concentrations produced by iNOS contributes to the development of tumors and tumorigenesis [85].

NO also influences glucose, glutamine, and lipid metabolism via PI3K/Akt signaling, which is the macrophage signal pathway involved in macrophage activation by responding to inflammatory signals [88] and which was found to be significantly activated in HCC cells [89]. Among the factors activated by the PI3K/Akt signaling pathway, the nuclear transcriptional factor Nrf2 modulates the expression of genes involved in glutamine metabolism [90], glutathione biosynthesis [91], and genes that promote the NADPH production for lipid metabolism [92] as well as the G6PDH gene.

Moreover, Akt promotes glycolysis and lipid biosynthesis by phosphorylation of ACLY and the activation of SREBP transcriptional factors [93] and suppresses the TCA cycle and oxidative phosphorylation [94]. L-citrulline, produced by iNOS, participates in L-arginine biosynthesis as a “loop” called “L-citrulline–NO cycle” to sustain macrophage activation. The enzyme argininosuccinate synthetase isoform 1 (ASS1) was found to be overexpressed in murine M1 macrophages [95], and its role could be considered the link between L-arginine metabolism and the TCA cycle to restore metabolites and reduce equivalents under inflammatory conditions.

In this context, L-citrulline reacts with L-aspartate via ASS1 to synthesize argininosuccinate, which is then converted by argininosuccinate lyase (ASL) into L-arginine plus fumarate. L-arginine is re-utilized for NO synthesis, while fumarate is transported into mitochondria, where it is utilized to sustain the interrupted TCA cycle by producing reducing equivalents (Figure 2).

M1 macrophages require a high amount of L-arginine, and it is possible that they also increase the de novo biosynthesis, as well as the L-arginine uptake. The de novo biosynthesis starts with L-glutamate, which is converted in ɣ-glutamyl phosphate by ɣ-glutamyl kinase, followed by a reduction catalyzed by ɣ-glutamyl phosphate reductase to L-glutamate-5-semialdehyde. This last compound participates in a reaction catalyzed by ornithine aminotransferase to produce ornithine. These reactions occur in mitochondria where ornithine is converted by ornithine transcarbamylase to L-citrulline, which is transported from mitochondria to the cytosol by the mitochondrial carriers of ornithine, ORC1 and ORC2, which transport substrates in exchange or in uniport. Both isoforms have a higher affinity for ornithine (Km 0.2 and 0.4 mM for ORC1 and ORC2, respectively) than for lysine or L-arginine (0.8 and 1.6 mM for ORC1 and ORC2, respectively) [96], although whether their expression level is increased in M1 macrophages or HCC cells remains to be investigated.

In M2 macrophages, the fate of L-arginine is different. In TAMs, also considered as M2 macrophages, an overexpression of ARG1 was found, which synthesizes ornithine, is metabolized in polyamine or proline, and is important for cell proliferation and collagen biosynthesis [97]. Through decarboxylation by ornithine decarboxylase (ODC), ornithine produces putrescine, which is then converted into spermidine and spermine by spermidine synthase (Spds) and spermine synthase (Spms), respectively. Alternatively, ornithine leads to proline by ornithine aminotransferase (OAT).

It is possible that when the L-arginine levels are very high, ARG1 contributes to regulating the L-arginine pool, promoting macrophage polarization toward the M2 phenotype. Ornithine, derived from ARG1, is transformed by mitochondrial ornithine transaminase in L-glutamate and then in glutamine to replenish the tumor environment of a carbon source (Figure 2). In this context, the role of L-aspartate and L-glutamate can be vital in sustaining the metabolic reprogramming of macrophages via malate/aspartate shuttle (MAS).

The MAS function could be relevant to maintaining redox balance and supplying substrates for biosynthesis, and it could be more relevant in M1 than in M2 macrophages. In cancer cells, as well as in HCC cells, MAS is crucial for tumor survival and proliferation, and some actors of MAS were found to be overexpressed [4].

In fact, MAS contributes to supplying L-aspartate from mitochondrial oxaloacetate derived from cytosolic malate. Considering that the TCA cycle is partially disrupted in both cancer cells and M1 macrophages [98], MAS can restore the redox balance between cytosol and mitochondria by allowing glycolysis to continue and by supplying reducing equivalents for the mitochondrial respiratory chain. The important players of MAS are the aspartate/glutamate mitochondrial carriers (AGC1 and AGC2) that transport L-aspartate from mitochondria in exchange with L-glutamate. Considering the kinetic characteristics of AGC1 and AGC2, both proteins show the same affinity for L-aspartate and L-glutamate (Km of 0.05 and 0.2 mM, respectively), but Vmax is four times higher for AGC2 than AGC1 [96], confirming that in normal condition, AGC2 is the main isoform of MAS. In HCC, HepG2 cell line, while AGC2 is downregulated, AGC1, which is normally absent in the liver, was found to be overexpressed, underlying its function beyond the one in the MAS pathway, particularly in biosynthetic pathways like nucleotide or amino acid biosynthesis for cell proliferation [99,100].

It was reported that in LPS-activated macrophages, both AGC1 and AGC2 mRNA levels are increased [101], leaving us to speculate that these mitochondrial carriers are relevant in the metabolic reprogramming of M1 macrophages.

#### 4.3.2. L-Glutamine Metabolism

Furthermore, the reprogramming of macrophage metabolism is supported by the metabolism of L-glutamine and L-glutamate; however, the precise function of L-glutamine in different macrophage phenotypes remains unclear. For example, in LPS-activated macrophages, L-glutamine is involved in the induction of HIF-1 [102]. On the contrary, it was demonstrated that α-ketoglutarate (αKG) derived from L-glutamine contributes to M2 activation [103].

In M1 macrophages, the L-glutamine catabolism is an important pathway to supply carbon and nitrogen sources. L-glutamine is converted via glutaminase in L-glutamate that can be considered as a metabolic cross point to produce αKG via L-glutamate dehydrogenase (GDH) to replenish TCA cycle or via L-aspartate transaminase for MAS function supporting mitochondrial function and bioenergetics (Figure 2). L-glutamate contributes to glutathione biosynthesis, which is relevant in M1 macrophages [102] and is important in modulating mitochondrial biogenesis in HCC [104]. In this context, glutamate carriers (GC1 and GC2), uniporters that transport L-glutamate across the mitochondrial inner membrane, could play an important role. Although GC1 mRNA is expressed at higher levels in all tissues compared to GC2 mRNA, GC1 shows a higher Km (5.18 mM) and Vmax for L-glutamate uniport compared to Km (0.26 mM) and Vmax of GC2, suggesting different roles for these isoforms in both basal and increased demands for L-glutamate [105].

In M1 macrophages, GC1 gene expression was found to increase [106], and we speculate that when the L-glutamate levels increase, GC1 may switch the L-glutamate metabolism towards biosynthesis, as GSH biosynthesis. L-glutamine biosynthesis is an interesting point in metabolic reprogramming observed in M2 macrophages. The enzyme glutamine synthetase (GS) plays a main role related to the M2 function, and its inhibition favors a switch from the M2 to the M1 phenotype in TAM [107]. In this context, macrophage L-glutamine synthesis could be relevant to sustain the growth of tumor cells that utilize L-glutamine as carbon sources to replenish the TCA cycle via glutaminase and GDH, which is overexpressed in many tumors as well as in HCC [104].

## 5. Metabolites of TCA Cycle and Macrophage Reprogramming

### 5.1. Citrate

In the last ten years, the cellular role of citrate has been reconsidered in light of its involvement in the metabolic reprogramming of many types of cells, including cancer and immune cells [108,109].

Citrate is produced in the mitochondria via citrate synthase (CS) and takes part in the TCA cycle, the main pathway for the bioenergetic, biosynthetic, and redox balance of cells. Furthermore, citrate can be transported from mitochondria to the cytosol in exchange with malate by CIC, the mitochondrial carrier of citrate, for the biosynthesis of fatty acids. Traditionally, in hepatocytes, the main role of citrate was related to lipid biosynthesis and glycolysis inhibition. However, in M1 macrophages and cancer cells, like HCC cells, citrate assumes a different function.

Despite the TCA cycle being interrupted at several points, CS and CIC were found to be overexpressed in aggressive cancer cells and M1 macrophages, respectively [4,110]. Consequently, mitochondrial citrate seems to be involved in the activation of other pathways that sustain the proliferation and growth of cancer cells and the inflammatory response of macrophages. In turn, citrate is exported by CIC, and in the cytosol, it is the substrate of ACLY to produce oxaloacetate (OXA) and acetyl CoA. OXA is transformed by malate dehydrogenase 1 (MDH1) into malate, which is transported into mitochondria through the MAS by regenerating NADH to produce ATP or by malic enzyme (ME), leading to NADPH for ROS and NO biosynthesis, which are important for both kinds of cells [41].

Acetyl CoA is the substrate for the biosynthesis of fatty acid, cholesterol, and other lipids and their derivatives, as well as arachidonic acid. However, acetyl CoA is used for protein acetylation as histones [41,111].

Interestingly, ACLY is overexpressed in M1 macrophages and can translocate into the nucleus to contribute to gene expression regulation via the acetylation mechanism [73], thereby confirming another interesting role of citrate in these conditions.

In many cancers, as well as lung adenocarcinoma and ovarian cancer, ACLY was found to be overexpressed [112,113]. Furthermore, analysis using Western blotting and qRT-PCR revealed that ACLY is upregulated in primary HCC tissues and related to the poor prognosis of HCC patients. Finally, the ACLY silencing led to the inhibition of the migration of HCC cells [114]. Based on these considerations, ACLY assumes an oncogenic role in HCC mediated by acetylation of proteins.

### 5.2. Alpha-Ketoglutarate, Succinate, and Fumarate

In the TCA cycle, αKG is produced by mitochondrial isocitrate dehydrogenase (IDH2). It was found that in many cancer cells, IDHs are mutated [115] and were downregulated in M1 macrophages [116]. Both conditions lead to a decrease in the enzymatic activity of IDHs to the first stop of the TCA cycle; consequently, isocitrate could be converted to citrate in a reverse TCA cycle but also itaconate via aconitate decarboxylase (ACOD1 encoded by IRG1 gene) in immune cells [117] or to oncometabolite D-2-hydroxyglutarate (D-2HG), which is exported in TME and may aid the TAM polarization and function in cancer cells [115]. In fact, elevated levels of D-2HG are measured in the serum of patients with IDH1/2 mutated cancer as intrahepatic cholangiocarcinoma [118].

As previously explained, the pool of αKG could be replenished via glutaminolysis, where L-glutamine is transformed to L-glutamate, which is then converted into αKG by GDH or aspartate transaminase. Interestingly, GDH was overexpressed in HCC cells [104] as well as in M1 macrophages, confirming the main role of αKG pool as a crossroad of metabolic signals in these conditions. The metabolite succinate is derived from succinate dehydrogenase (SDH), a component of both the TCA cycle and the mitochondrial respiratory chain’s Complex II. In M1 macrophages, SDH activity is altered, and succinate accumulation leads to ROS generation and HIF-1α stabilization, contributing to inflammatory response induction [119]. In cancer cells, succinate accumulates because of SDH mutation or reduced expression. Succinate supports the stabilization of HIF-1α, contributing to tumor alteration. Furthermore, in HCC cells, SDH is reduced, whereas succinate levels are increased with poor prognosis [120].

It is unclear whether M1 macrophages and cancer cells secrete succinate into the microenvironment. Recently, it was demonstrated that extracellular succinate upregulates M2 macrophage gene expression and downregulates that of M1 macrophage genes, thus shifting the polarization towards M2 macrophage. Nonetheless, the membrane succinate transporter, SUCNR1, was overexpressed in M2 macrophages and regulated the anti-inflammatory response of macrophages in obesity [121]. Currently, the role of succinate in different macrophage phenotypes and cancer cells is not fully understood.

Another interesting immunometabolite is fumarate, which is derived into mitochondria from succinate by SDH and converted into L-malate by fumarate hydratase (FH) or in the cytosol by aspartate-argininosuccinate shunt by ASS1 and ASL enzymes. In FH-deficient cells, the concentration of fumarate is in the millimolar range, and fumarate could accumulate in different cellular compartments, such as mitochondria, cytosol, and nuclei, as well in the extracellular environment [122].

Recently, in macrophages stimulated with LPS, an increase in cytosolic levels of fumarate was observed due to an increase in ASS1 expression and a decrease in FH expression. Furthermore, the inhibition of FH leads to an increase of inflammatory cytokine as TNF-α, causing a metabolic rewiring and a release of nucleic acids, which promote inflammation [117,123].

In cancer cells, FH was found to be mutated, causing a decrease in activity, with an accumulation of fumarate, which can lead to inactivation of prolyl hydroxylase (PHD), TET inhibition, and protein succination. The inactivation of PHD leads to HIF-1α stabilization, whereas TET inhibition is important to DNA/histone methylation and to gene expression to cellular growth and proliferation [4].

Finally, post-translational modification succination was found in many proteins, as well as Kelch-like ECH-associated protein1 (KEAP1), iron regulatory protein 2 (IRP2), and aconitase (ACO2) [122]. These considerations indicate that fumarate levels are important to immune and cancer cells to address metabolic changes.

## 6. Metabolic Crosstalk Between HCC Cells and TAMs

In TME, TAMs are the major component of the immune cell population.

In light of the previous metabolic considerations and the differences between M1 and M2 macrophages, the metabolic shift can be considered the checkpoint that helps the macrophages respond to environmental demands, with a macrophage polarization between M1 and M2 as inflammatory and anti-inflammatory immune cells, respectively. In fact, when there is an inflammatory stimulation, macrophage metabolism shifts toward the M1 phenotype, whereas when the inflammation has to end, the metabolism is like the M2 phenotype.

We can speculate that there is a continuous balance between the M1 and M2 phenotypes, and turning these on and off is governed by metabolism.

Cancer cells behave like M1 macrophages and send messages to shift all macrophages into the M2 phenotype, in turn, to restore a new “macrophage” balance. In fact, most TAMs are described as M2 macrophages [124].

What are the signals sent by cancer cells to shift macrophage metabolism?

There is not one only answer. We can speculate that many mediators could contribute to turning off the M1 aggressive response to change in an unaggressive behavior like M2 macrophages. For example, NO is one of the inflammatory mediators in M1 macrophages [125]. However, NO was found to promote M2 polarization in some cells [126] by highlighting its complex role in macrophage remodeling. iNOS is found to be overexpressed in HCC cells [85], and, considering that it is a diffusible molecule, its effect propagates in TME [84], contributing to increased NO concentration and, probably, to shift M2 polarization.

PGE2 is another inflammatory mediator related to M1 macrophages because PGE2 production is increased in inflammation, promoting M1 polarization [127]. However, recent studies in atherosclerosis have linked PGE2 to M2 activation and M1 inhibition, and this polarization seems mediated by different regulatory transcriptional factors such as CREB and NF-κB [128].

PGE2 levels are reported to be increased in various cancer types [129], and HCC cancer cells show a high level of PGE2 that promotes HCC progression [130].

In light of these considerations, we hypothesize that PGE2 levels also promote M2 polarization, helping to resolve the “inflammatory-like condition” of the environment.

Furthermore, metabolites such as citrate, succinate, and fumarate contribute to the interplay between cancer and immune cells. These metabolites accumulate in many kinds of cancer cells as well as in M1 macrophages.

Therefore, cancer cells resemble M1-like cells, while TAMs become M2-like types, creating a complex pro-inflammatory and anti-inflammatory balance.

## 7. A Clinical Viewpoint on Therapeutic Interventions

The treatment of HCC and the management of advanced disease stages have changed during the last two decades. HCC is efficiently treated by liver transplantation and local radiofrequency ablation in the early stages, but many patients with advanced HCC may not benefit from these therapies [131,132].

As the knowledge about the interplay between tumoral and non-tumoral cells in TME is increased, many new approaches for the prevention and treatment of HCC have been developed, and metabolic targets, together with the immunotherapeutic strategy, represent a valid opportunity to explore.

The immunotherapeutic strategy, focusing on macrophage shift toward the M1 phenotype, together with metabolic enzyme modulation to address this reprogramming, is a promising approach that aids in developing novel treatments.

Currently, the therapy for advanced-stage patients includes immune checkpoint inhibitors and monoclonal antibodies. The combination of atezolizumab, the anti-programmed cell death ligand 1 (PDL1) antibody, with bevacizumab, the anti-VEGF antibody, has been approved for the treatment of patients with unresectable liver cancer [133]. Furthermore, nivolumab, the anti-programmed cell death protein 1 (PD-1), has been proposed for the treatment of advanced HCC patients who have been treated with sorafenib (ID NCT02576509), and the combination of Ipilimumab, the anti-cytotoxic T-lymphocyte antigen 4 (CTLA-4), and nivolumab suggests an increased response rate for advanced HCC (ID NCT03510871).

Considering the alteration of lipid metabolism in TME, several studies have demonstrated that HCC incidence decreased in patients treated with statins compared to those untreated [134]. The clinical study “Effects of Statin on Hepatocellular Carcinoma Recurrence After Liver Transplantation” (ID NCT03490461) investigated the effect of statin therapy on the incidence of HCC recurrence following transplantation. A new clinical trial (ID NCT05028829) will be carried out to examine how atorvastatin affects the risk of HCC. Statins are inhibitors of 3-hydroxy-3-methyl-glutaryl CoA reductase, the rate-controlling enzyme of cholesterol biosynthesis. Furthermore, they have been related to antiangiogenic, antiproliferative, and immunomodulatory effects that decrease HCC progression [135]. The NF-kB pathway, protein kinase B inhibition, or a reduction in pro-inflammatory cytokines have all been implicated in the anti-tumor effect of statins [136].

Lactate metabolism targeting may be an intriguing approach for treating HCC. Nowadays, there are no clinical drugs, but promising candidates are inhibitors of MCT1 and MCT4. Interestingly, AZD0095 is a selective inhibitor of MCT4 tested on cancer cell lines with overexpression of MCT4, as well as HCC cells [137].

Cyclooxygenase 2 (COX2) is overexpressed in different cancers, including HCC, and is associated with poor prognosis [138]. COX2 leads to the synthesis of PGE2 that, among the inflammatory mediators, promotes multiple mechanisms underlying tumor initiation and progression [139]. Evidence indicates that aspirin and non-steroidal anti-inflammatory drugs (NSAIDs), which inhibit PGE2 production, may help prevent tumor initiation and influence tumor progression [140,141]. Celecoxib, a selective COX2 inhibitor, suppresses HCC cell growth and invasion alone [142] or in combination with other drugs [143,144,145]. Since HCC patients with high COX-2 expression exhibit significantly higher numbers of M2 macrophages in TME, which impairs T cell cytotoxicity [146], the treatment with Celecoxib diminishes the suppressive impact on CD8+ T cells by reducing M2-like TAMs. This enhances the effectiveness of T cell-based immunotherapy and improves the prognosis for HCC patients [146].

## 8. Future Perspectives and Conclusions

The complex network of TME, in which TAMs play an intriguing role, dynamically regulates the growth, development, and invasion of HCC cells. TME, in turn, may affect the polarization and metabolic features of macrophages, impacting their function. Interestingly, HCC cells secrete signaling molecules, metabolites, and growth factors that induce the shift of TAMs toward the M2 phenotype [147].

High plasticity is a hallmark of macrophages, which can remodel their phenotype according to microenvironmental signals. It is well-known that metabolic reprogramming plays a critical role in driving macrophage and cancer cell phenotype.

The heterogeneity of TAMs makes it very difficult to understand this interaction. TAMs arise from different sources and, by responding to different microenvironments, lead to tumor growth by multiple mechanisms [148], thus playing a crucial role in promoting HCC progression.

Furthermore, the molecular pathogenesis of HCC, with a particular emphasis on variability among patients, has been investigated [149]. The Barcelona-Clinic Liver Cancer (BCLC) classification with stage definition [150] and other HCC subtype classifications with distinctive clinical, molecular, and phenotypic characteristics [151] underline that HCC is a highly heterogeneous disease.

In light of this complex landscape, it is very hard to understand the biology of HCC; consequently, targeting TAMs and their metabolic dysregulation may enhance the effectiveness of anti-cancer therapy. However, much remains to be discovered regarding the remodeling of macrophages and their interplay with tumor/HCC cells.

The increased knowledge about new metabolic targets of cancer cells and macrophages could aid in developing combined approaches for cancer therapy, in which metabolic reprogramming could be one of the targets for restoring the balance between cells.

Therefore, future investigations will be crucial in understanding the intricate relationship between metabolic changes and the phenotype of TAMs.

## Figures and Tables

**Table 1:** Macrophage types and subtypes.

	M1	M2				Mox	M4	Mhem
		M2a	M2b	M2c	M2d			
Stimuli	IFN γ LPS IRF1 IRF5 IRF8	IL-4 IL-13	IL-1β IL-1R IC + TLR agonists	IL-10 TGF-β Glucocorticoids	IL-6, LPS+ A2R agonists	OxPL	CLCX4	Hb

Abbreviations: IFNγ, interferon γ; LPS, lipopolysaccharide; IRF, interferon regulatory factors; IL, interleukin; IC, immune complexes; A2R, A2 adenosine receptor; TGF-β, transforming growth factor β; OxPL; oxidized phospholipids; CXCL4, chemokine ligand 4; Hb, hemoglobin.
